# Comparison of initial oral microbiomes of young adults with and without cavitated dentin caries lesions using an *in situ* biofilm model

**DOI:** 10.1038/s41598-018-32361-x

**Published:** 2018-09-18

**Authors:** Stefan Rupf, Cedric C. Laczny, Valentina Galata, Christina Backes, Andreas Keller, Natalia Umanskaya, Arzu Erol, Sascha Tierling, Christina Lo Porto, Jörn Walter, Jasmin Kirsch, Matthias Hannig, Christian Hannig

**Affiliations:** 1 Clinic of Operative Dentistry, Periodontology and Preventive Dentistry, Saarland University Medical Center, Homburg, Germany; 2 Chair for Clinical Bioinformatics, Saarland University, Saarbrücken, Germany; 3 Faculty of Natural Sciences and Technology, Department of Genetics/Epigenetics, Saarland University, Saarbrücken, Germany; 4 Policlinic of Operative and Pediatric Dentistry, Medical Faculty Carl Gustav Carus, TU Dresden, Dresden, Germany

## Abstract

Dental caries is caused by acids released from bacterial biofilms. However, the *in vivo* formation of initial biofilms in relation to caries remains largely unexplored. The aim of this study was to compare the oral microbiome during the initial phase of bacterial colonization for individuals with (CC) and without (NC) cavitated dentin caries lesions. Bovine enamel slabs on acrylic splints were worn by the volunteers (CC: 14, NC: 13) for *in situ* biofilm formation (2 h, 4 h, 8 h, 1 ml saliva as reference). Sequencing of the V1/V2 regions of the 16S rRNA gene was performed (MiSeq). The relative abundances of individual operational taxonomic units (OTUs) were compared between samples from the CC group and the NC group. Random forests models were furthermore trained to separate the groups. While the overall heterogeneity did not differ substantially between CC and NC individuals, several individual OTUs were found to have significantly different relative abundances. For the 8 h samples, most of the significant OTUs showed higher relative abundances in the CC group, while the majority of significant OTUs in the saliva samples were more abundant in the NC group. Furthermore, using OTU signatures enabled a separation between both groups, with area-under-the-curve (AUC) values of ~0.8. In summary, the results suggest that initial oral biofilms provide the potential to differentiate between CC and NC individuals.

## Introduction

Dental caries is a major health problem. Recent data demonstrate its high relevance for both the global prevalence of chronic asymptomatic caries as well as for the incidence of acute-caries associated tooth pain^1,2^. Dental caries is a process, which is mainly determined by lifestyle and can be delayed or even prevented by measures of oral hygiene and the use of fluorides^3^. However, even in developed countries young adults still display a remarkably high prevalence of decayed and filled tooth-surfaces^4,5^.

The caries process leads to the destruction of enamel and dentin by acids of microbial origin formed in dental biofilms^6^. Biofilm development is characterized by several typical stages irrespective of caries activity. These stages include the formation of an abacterial acquired pellicle, adhesion of saliva-borne bacterial early colonizers, and the formation of an extracellular matrix^7,8^. Finally, detached biofilm components are taken up by saliva and form the basis for the settlement on further surfaces. The cariogenicity of the biofilms can be influenced by diet and systemic diseases^6^. A diet rich in carbohydrates potentially leads to an increase of aciduric and acidogenic bacteria in biofilm and in saliva^9^. The availability of mono- and disaccharides in the food leads to a short-term decrease of the pH in plaque. This acidic attack dissolves calcium hydroxyapatite from the tooth’s hard substance. A high frequency of such acidic attacks over a prolonged period leads to a transition of biofilms from a healthy to a pathogenic state^8^, accelerating the dynamics of the caries process and followed by the formation of cavities^10,11^. Since caries is a process strongly influenced by microorganisms, the analysis of the microbiomes of biofilms in all stages and in saliva is crucial to further advance the understanding of this process and to provide tools for caries prediction and prevention^6,12^.

Biofilm *in situ* models offer a promising and standardizable option for obtaining biofilm samples over strictly defined periods of time^7,13^. These *in situ* models can be used to investigate initial bioadhesion processes, basic microbiological and molecular genetic questions, as well as the influences of food and oral hygiene products^13–15^. Depending on the requirements, glass, polymers, dental materials or dental hard substances can be used as the basic substrate^13,16–18^. Bovine enamel is particularly suitable for simulating the natural formation of biofilm on tooth enamel. The cattle are BSE-free and their meat is approved for human consumption. This procedure avoids the use of human enamel and appears more tolerable for many test persons. Furthermore, it is much easier to produce samples of homogeneous quality.

Conventional microbiology approaches have been used successfully and repeatedly for the analysis of the oral microbiome^19–21^. In addition, recent advances in culture-independent approaches, e.g., using high-throughput random shotgun sequencing (Next Generation Sequencing, NGS), provide improved turn-around times, reduced costs, and the potential to resolve hitherto unculturable microorganisms^22^. Meanwhile, about 700 prokaryote species have been detected in the oral cavity^23^. A comprehensive overview of oral bacterial taxa is available in the human oral microbiome database (eHOMD)^23,24^. NGS is also used for specific questions on the composition of the oral microbiome in healthy and diseased individuals^25–27^. With NGS methods, differences in the oral microbiome composition of individuals with severe, as well as those with moderate caries, were identified^28–33^. The general process of initial colonization of bacteria on tooth surfaces has been investigated *in vivo* and *in situ* with conventional approaches^34–39^. However, only few recent studies have characterized the microbiome during initial bacterial colonization in healthy adults *in vivo* or *in situ* using high-throughput sequencing approaches^40–42^. Therefore, the aim of the present study was to evaluate and to compare the microbiome of the initial phase of bacterial colonization on an *in situ* enamel biofilm model systematically after 2 hours, 4 hours and 8 hours for young adults with and without cavitated caries for the first time.

## Results

### Subjects

Groups of individuals without clinical signs of current caries activity (NC; 13 individuals) or with cavitated caries (CC; 14 individuals) were comparable with respect to their age (NC: median age 25 y, min-max: 19 to 33 y; CC: median 25 y, min-max 20 to 30 y), oral hygiene (approximal plaque index: NC: 61.6 ± 29.4%, CC: 70.8 ± 27.7%) and oral inflammation (modified sulcus bleeding index: NC: 35.2 ± 24.2%, CC: 44.6 ± 23.8%). All subjects showed physiological salivary flow rates of >0.25 ml saliva per minute. NC individuals presented no open decayed surfaces whereas CC individuals had a mean of 4.4 ± 1.8 cavitated carious surfaces reaching into the dentin. No open caries lesions were observed in the NC group (Supplemental Table 1).

### General sequence data

**Figure 1:**
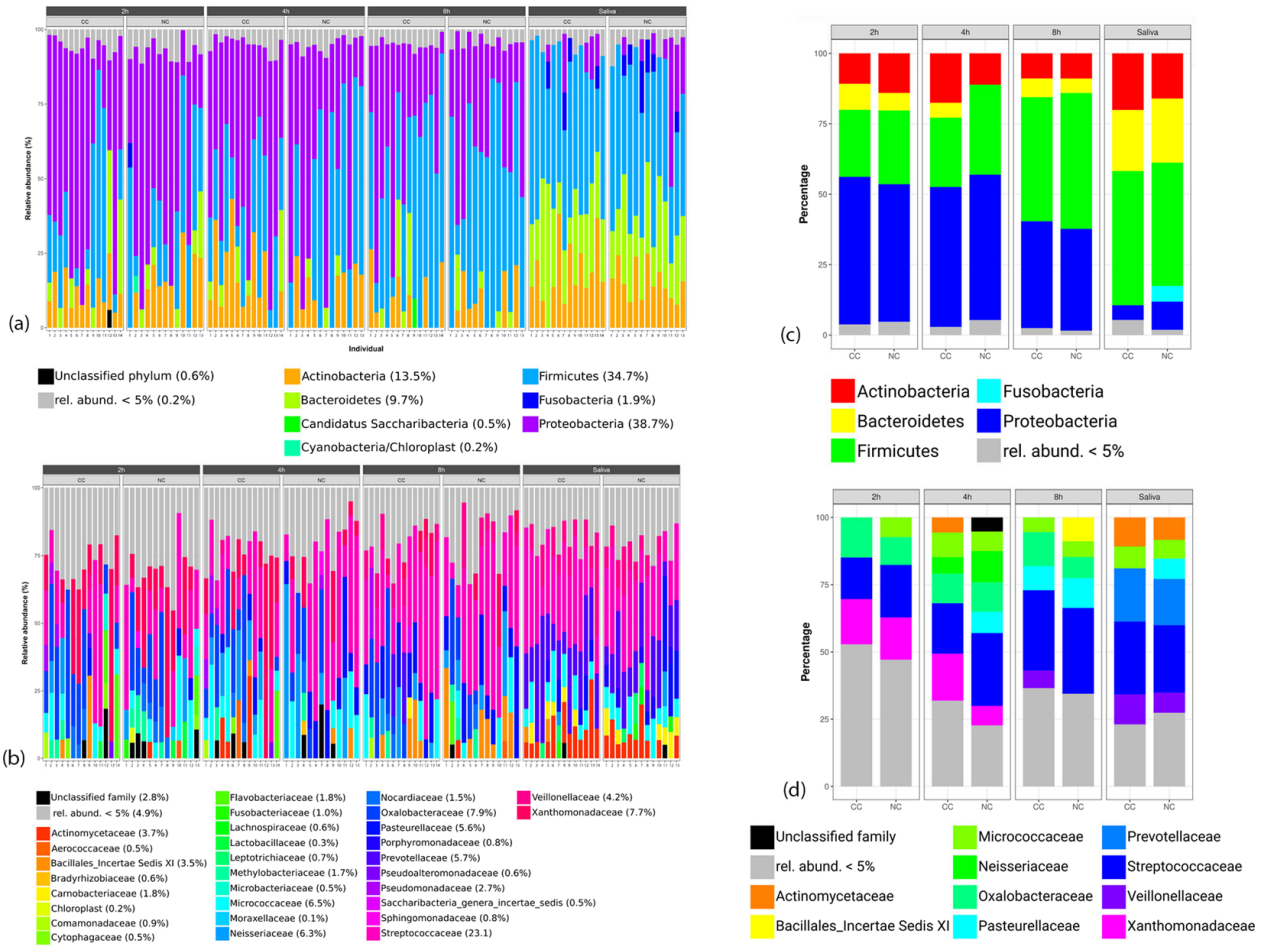
Relative abundances of individual taxa per sample at phylum (**a**) and family (**b**) level, and summarized per indication at phylum (**c**) and family (**d**) level. The abundances were computed over all OTUs before filtering out OTUs present in controls. Samples were grouped by indication (CC: cavitated caries, NC: no cavitated caries) and time point (2 h-, 4 h- and 8 h-biofilms, and saliva) in (**a**) and (**b**); in (**c**) and (**d**), the indication is shown per time point. Taxa with a relative abundance below 5% per sample (**a**,**b**) and indication (**c**,**d**) were grouped into one category (“rel. abund. <5%”). In panels (a) and (b), the relative taxon abundance across all non-control samples and OTUs is shown in brackets next to the taxon name in the legend. It was computed as the sum of OTU counts corresponding to the respective taxon divided by the total OTU count across all non-control samples.

**Figure 2:**
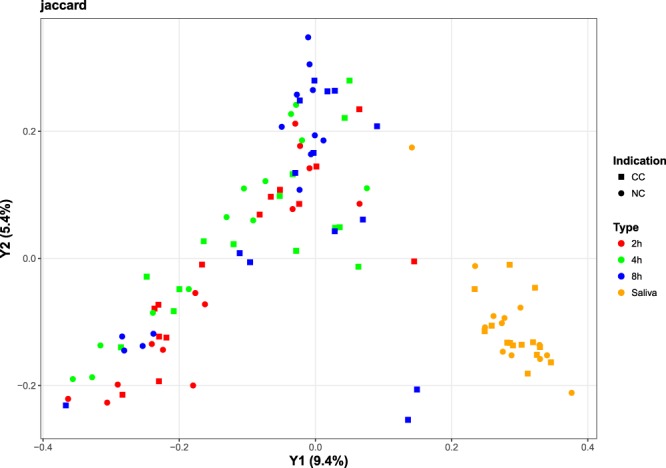
PCoA-based ordination of the Jaccard distance matrix. Colors reflect the sample type (2 h, 4 h, 8 h, and saliva) and shapes reflect the CC and NC groups. The percentage of variation explained is included in the axis labels.

**Figure 3:**
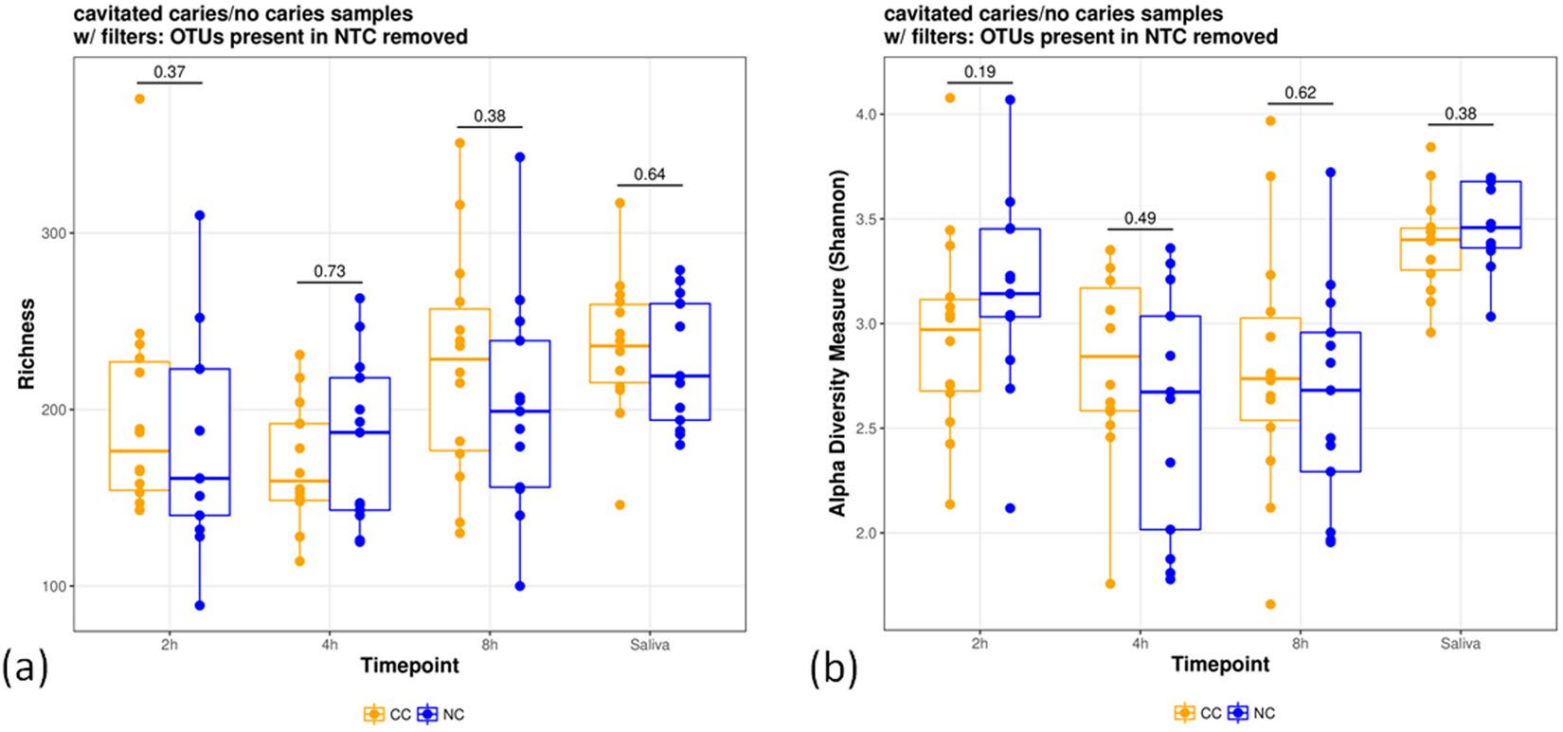
Richness (**a**) and alpha diversity (**b**) measures of different time points (2 h-, 4 h-, and 8 h-biofilms) and for saliva and indications (CC: cavitated caries, NC: no cavitated caries). OTUs present in the NTC were removed and richness and Shannon’s alpha diversity measures are shown. The individual points represent individual diversity estimates.

A total of 2,192,964 sequences were obtained from all 81 biofilm samples (2 h, 4 h, and 8 h) and from all 27 saliva samples after processing using LotuS (Supplemental Fig. S1). Additionally, 160,542 sequences were found in the DNA preparations of non-orally exposed enamel specimens (enamel control) or water (PCR control), and 9,350 sequences in the non-template control (NTC). OTUs were defined at the 97% identity level and 1,875 OTUs remained after discarding OTUs present in any of the controls (Supplemental Table 2). The identified OTUs were derived from members of the *Proteobacteria* (28%), *Bacteroidetes* (15%), *Firmicutes* (15%), *Actinobacteria* (10%), *Fusobacteria* (3%), *Candidatus Saccharibacteria* (2%), and *Parcubacteria* (2%) with other phyla covering less than 2% of the remaining OTUs according to taxonomic assignments created by LotuS. Nineteen percent of OTUs could not be assigned to a phylum. An overview of the taxonomic composition of OTUs in all samples as well as of the excluded OTUs present in controls is provided in the supplements (Supplemental Fig. S2a,b). Furthermore, per-sample compositions on phylum and family levels over all OTUs are presented in Fig. 1a,b. A high heterogeneity of the individual microbiomes is apparent (Fig. 1a,b). While a pronounced difference can be observed for the saliva samples compared to the biofilm samples, the biofilm samples are mutually overlapping (Figs 1c,d and 2, Supplemental Fig. S3a,d). Performing the ordination only on biofilm samples, i.e., excluding the saliva samples, did not lead to a markedly improved separation of the biofilm time points (Supplemental Fig. S4). Moreover, no statistically significant differences of the alpha-diversity nor of the richness were found when comparing the CC and NC groups per time point or in saliva (Wilcoxon-Mann-Whitney test, alpha = 0.05, Fig. 3a,b).

### Statistical pairwise comparisons of individual OTUs

**Figure 4:**
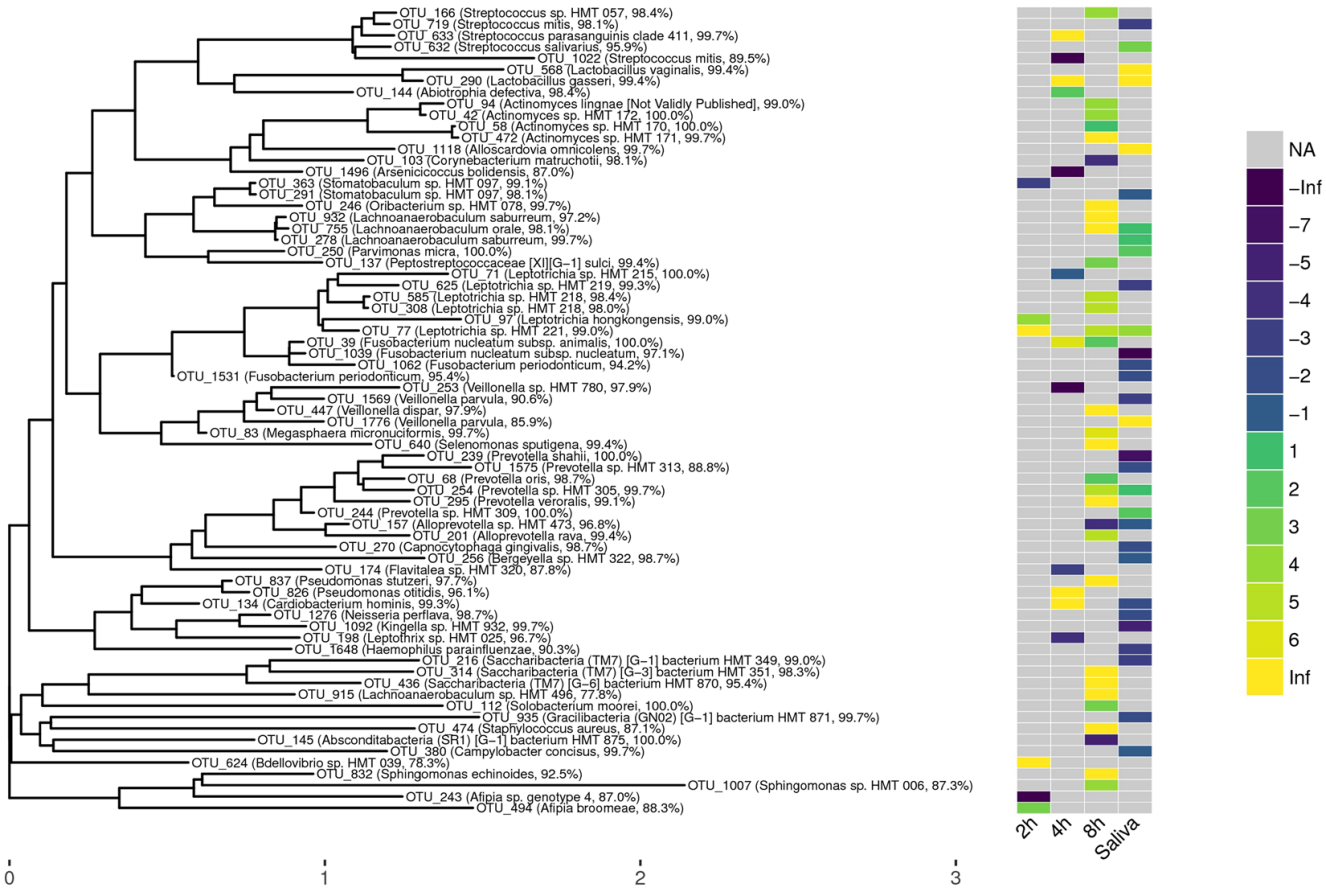
Phylogenetic OTU tree created by LotuS with taxonomic assignment from the eHOMD database. Only OTUs with significantly different relative abundance are depicted with a heat map showing the log2(fold change) within the respective groups used for comparison. The values were highlighted as missing (“NA”), or colored w.r.t. their rounded value. A positive log fold-change indicates a higher relative abundance in the cavitated-caries group, a negative log fold-change stands for higher abundances in the no cavitated-caries group. Match identities according to eHOMD are given following the taxonomic name.

While the overall compositions at the phylum level as well as at other taxonomic levels were largely consistent between samples from individuals with cavitated caries and individuals without cavitated caries, individual OTUs demonstrate statistically significant differential relative abundances when comparing NC to CC samples (Fig. 4). Altogether, independent of the time point, statistically significant differences were found for 71 distinct OTUs (Fig. 4, Supplemental Table 3). At the 2 h time point, six significantly different OTUs were identified (genus according to eHOMD: 2x *Leptotrichia*, *Stomatobaculum*, 2x *Afipia*, and *Bdellovibrio*). At the 4 h time point, 12 significant hits were found (2x *Streptococcus*, *Lactobacillus*, *Abiotrophia*, *Arsenicococcus*, *Leptotrichia*, *Fusobacterium*, *Veillonella*, *Flavitalea*, *Pseudomonas*, *Cardiobacterium*, and *Leptothrix*). Moreover, 31 significant hits were detected at the 8 h time point (*Streptococcus*, 4x *Actinomyces*, *Corynebacterium*, *Oribacterium*, 3x *Lachnoanaerobaculum*, *Peptostreptococcaceae*, 3x *Leptotrichia*, *Fusobacterium*, *Veillonella*, *Megasphera*, *Selenomonas*, 3x *Prevotella*, 2x *Alloprevotella*, *Pseudomonas*, 2x *Saccharibacteria*, *Solobacterium*, *Staphylococcus*, *Absconditabacteria*, and 2x *Sphingomonas*). Finally, for the saliva samples, 30 significant hits were found additionally (2x *Streptococcus*, 2x *Lactobacillus*, *Alloscardovia*, *Stomatobaculum*, 2x *Lachnoanaerobaculum*, *Parvimonas*, 2x *Leptotrichia*, 3x *Fusobacterium*, 2x *Veillonella*, 4x *Prevotella*, *Alloprevotella*, *Capnocytophaga*, *Bergeyella*, *Cardiobacterium*, *Neisseria*, *Kingella*, *Haemophilus*, *Saccharibacteria*, *Gracilibacteria*, and *Campylobacter*). While individual OTU hits occurred on more than one time point and with consistent direction-of-change (i.e., more or less abundant), e.g., OTU 77 (*Leptotrichia* sp. according to eHOMD) and OTU 254 (*Prevotella* sp. according to eHOMD) were more abundant in the CC groups of the 8 h time points and saliva, no single OTU was found to have significantly different relative abundance in all groups or time points (Supplemental Fig. S5). Furthermore, it was observed that the majority of OTU hits for the 8 h samples showed a positive log fold-change. Thus, they were more abundant in the CC group, whereas the majority of the OTU hits for the saliva samples showed a negative log fold-change.

**Figure 5:**
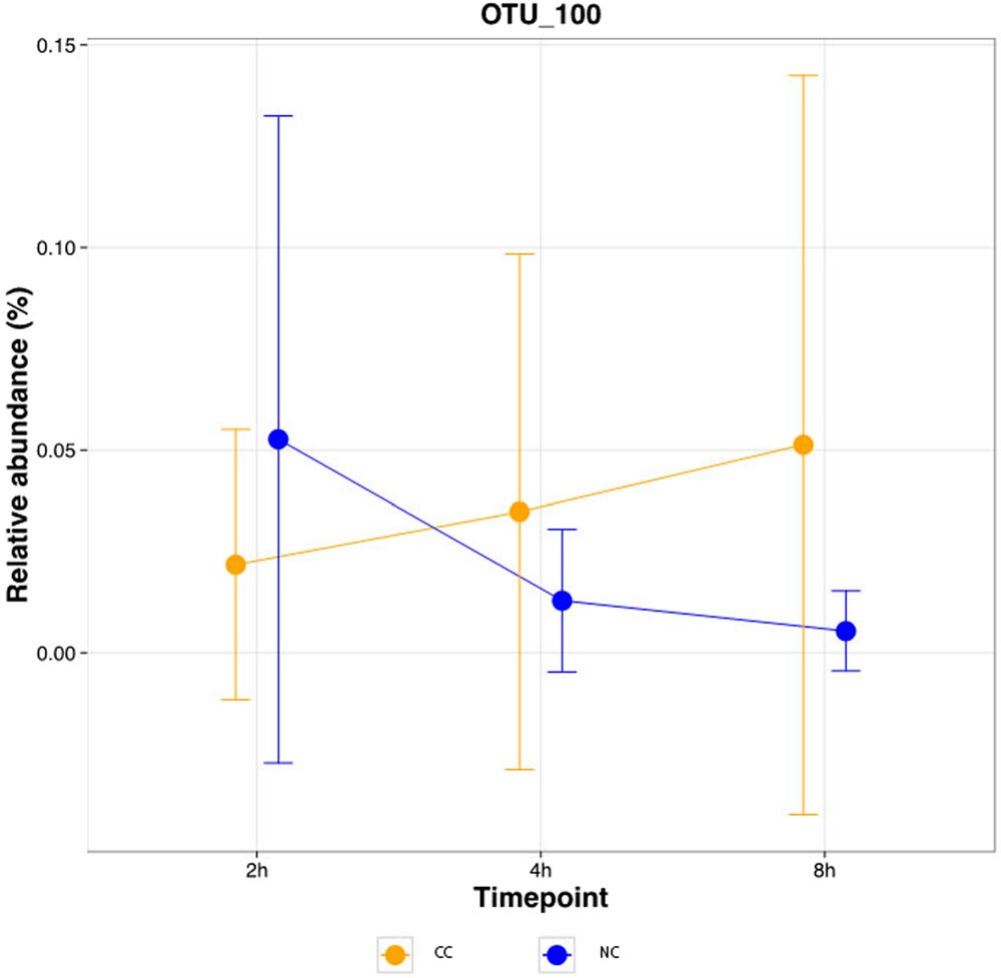
Relative OTU abundance over time per indication for an exemplary OTU. The mean +/− standard deviation of OTU 100 (*Moryella sp*. according to LotuS, *Stomatobaculum longum* according to eHOMD); absolute difference of the correlation of the mean values = 2 are shown.

The mean relative OTU abundances were additionally compared over time between individuals with and without cavitated caries. The absolute difference in the correlation values for the groups was computed, possibly reaching a maximal value of 2, showing strongly contrasting behavior over time (exemplary OTU 100 in Fig. 5). A total of 109 OTUs were found to have an absolute difference in their correlation values greater or equal to 1.5 (Supplemental Table 4).

### OTU signatures for specimen classification

**Figure 6:**
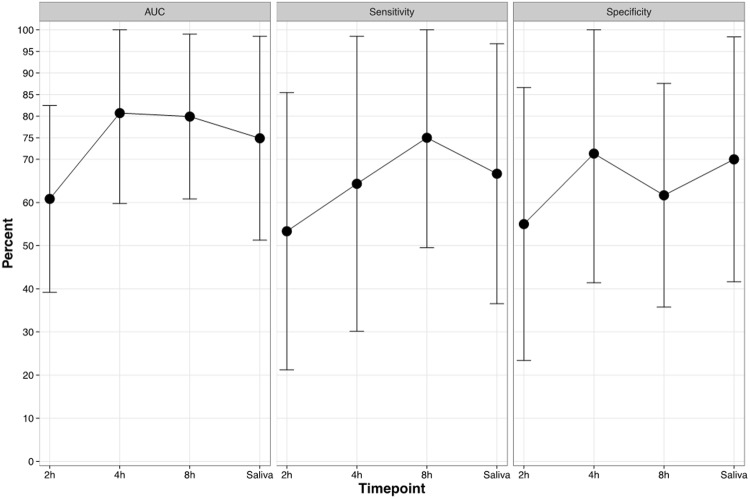
Classification performances. The cross-validated (5-fold, 10 repetitions) means +/− standard deviations (capped at 100%) of AUC, sensitivity, and specificity are shown per time points for the classification of samples as “cavitated caries”.

In addition to the study of individual OTUs, OTU signatures, i.e., combinations of individual OTUs, were used to train *in silico* classifiers for each time point and the saliva samples. The classification of samples obtained at 2 h exhibited the lowest mean values of AUC, sensitivity, and specificity (Fig. 6). At 4 h and 8 h, the mean AUCs were ~0.8. While the specificity was higher for the earlier time point, the later time point showed higher sensitivity in identifying samples from the CC group. The mean sensitivity for the initial biofilm samples increased over time, reaching its maximum at 8 h. In contrast, the 4 h samples showed the highest mean specificity values compared to all other samples (Fig. 6). The classification performance of saliva samples was below that of the 4 h and 8 h time points in terms of AUC, and between 4 h and 8 h in terms of sensitivity and specificity.

## Discussion

For the first time, the present *in situ* study evaluated and compared initial bacterial colonization on a biofilm model in adult individuals of different caries status using next generation sequencing. The results offer new information for basic research in oral microbiology concerning the process of bacterial colonization on dental enamel. In this context oral microbiomes were investigated, which consisted of biofilms formed over the periods of 2 h, 4 h and 8 h. The present *in situ* biofilm model enables the reproducible placement of identical biofilm-free enamel specimens in different individuals. This biofilm model thus overcomes the limitations of obtaining biofilms from natural tooth surfaces, as a well-defined and reproducible cleaning of tooth surfaces is only possible with great effort. Bovine enamel provides comparable properties as human enamel with respect to bioadhesion^18^. Tooth enamel from extracted teeth is of an unknown age. Furthermore, it is difficult to prepare larger samples from human teeth. The limitations of the *in situ* biofilm model are possible DNA contaminations from human salivary bacteria or from cattle oral microbiota. Saliva contamination was minimized by rinsing the specimens after removal from the oral cavity. Bacterial contamination from the bovine enamel itself was controlled by removing of all dentin and extensive cleaning of the enamel samples and by excluding all OTUs contained in control samples. It should also be noted that the NTC and the other no-biofilm controls showed contaminations. The corresponding OTUs were also systematically removed^43^.

The biofilms investigated in the present study represent typical oral microbiomes^14,25,27–33^. The 2 h and 4 h biofilms displayed high similarity; for the 8 h biofilms in particular the proportion of *Firmicutes* increased from 15% to 19%. Larger differences can be observed in comparison to the saliva samples. Here, the *Firmicutes* attained a share of 35%. The proportion of *Bacteroidetes* was 35% compared to 15% in biofilms. The relative abundance of *Bacteroidetes* was 35%. The proportion of *Fusobacteria*, *Candidatus Saccharibacteria* and *Spirochaetes* also increased, while the proportion of proteobacteria in biofilm samples dropped from more than 30% to 9% in saliva. A comparison of the microbiomes of the NC groups and the CC groups revealed remarkable differences. While in the NC group non-cariogenic taxa were significantly more frequent relatively, in the CC group members of the cariogenic microbiome were significantly more frequent with *Actinomyces*, *Lachnoaerobaculum* from the biofilms and *Lactobacillus* and *Lachnoaerobaculum* from the saliva, which is in line with previous reports^33^. Currently, caries risk prediction is mainly based on the inspection of existing caries lesions. This study presents a standardized *in situ* biofilm model which allowed, albeit not perfectly, to differentiate between young adults with and without cavitated caries using microbial signatures of early dental biofilms. Thus, this model represents a compelling new means for caries risk assessment, providing predictive readouts after 4 h or 8 h.

No universal biomarkers for caries prevalence could be detected herein, i.e., no single OTU was statistically significantly more or less abundant in the CC group compared to the NC group at all three time points (2 h, 4 h, 8 h) and saliva. This result is in accordance with the current state of knowledge and indirectly confirms the extended ecological plaque hypothesis, according to which aciduric and acidogenic bacteria gain a quantitative overweight in the biofilm under certain conditions, triggering a progression of the caries process^44^. Interestingly, hits in the 8 h samples were dominated by OTUs that were more abundant in CC samples, while the inverse was observed for the saliva samples, i.e., most OTU hits were more abundant in NC samples. As a clinical consequence resulting from this study, it might be useful to combine information obtained from microbiome samples of 8 h biofilms and of saliva to improve caries risk determination in the future.

The present results on OTU signature-based classifiers (instead of individual OTUs) demonstrated that, while the mean AUC values were between 75% and 80% (4 h, 8 h, saliva), and the sensitivity and specificity values increased over time, pronounced standard deviations could be observed. In future studies, a long-term follow-up of these moderate carious lesions presenting individuals should be carried out to link the oral microbiome compositions to the long-term development of caries lesions.

The use of a culture independent, 16S rRNA gene-based NGS approach limited our analyses to taxonomic assignments at the genus-level. However, individual species or even strains are expected to play crucial roles in the caries process. Thus, additional taxonomic analysis was performed using the eHOMD database providing species level resolution when possible (Supplemental Table 2, Fig. 4). However, considering the OTUs with significantly different relative abundances between the groups with and without cavitated caries, it is important to note that some of these OTUs have an assignment with identity percentage below 98.7% considered as gold-standard threshold for species classification^43^. Moreover, we want to emphasize that the taxonomic information is not used in our analyses which were all performed on OTU level regardless of any taxonomic assignment and its confidence.

Future studies, using metagenomic sequencing rather than amplicon sequencing, are expected to offer increased taxonomic resolution as well as to provide functional information which could be used for metabolic reconstructions. Another limitation of our study is the low biomass which is expected in the 2 h samples and, albeit to a much lesser extent, in the 4 h specimens. In this context, the sensitivity of the employed molecular approach bears challenges to be considered, e.g., contaminant DNA in the preparation kits or from environmental sources especially in the case of low biomass samples^45^. Though we attempted to remove potential contamination by strictly discarding all OTUs present in negative controls some contaminating sequences may still be present. Concretely, the phylum Cyanobacteria/Chloroplast is found in a single sample for the 2 h time point with a relative abundance >= 5% before filtering out OTUs present in controls (Fig. 1a). This indeed represents very likely a contamination. In any case, no chloroplast-related OTU was found to be statistically significantly differentially abundant. Whereas the 8 h biofilms seem to be preferable in order to limit the impact of possibly contaminating sequences, the 4 h biofilms could be considered as suitable only to a certain extent.

The present study confirms the results of a previous paper^46^ where pellicle enzymes had been investigated in the same subjects. Activities of human enzymes in the pellicle layer were of high similarity in both (CC and NC) groups. However, there was a tendency towards higher levels of bacterial glycosyltransferase D GTFD in subjects with open caries lesions. This finding is in accordance with the present findings with respect to differentially abundant taxa^46^.

In summary, by using a standardized *in situ* biofilm model and controlling for potentially confounding factors during 8 h of biofilm formation, we were able to detect differences in the oral microbiome compositions between individuals with and without cavitated caries. Moreover, when combined with standardized sample processing and sequencing procedures, the herein presented approach could be used to predict caries risk based on the composition of the oral microbiome. While further research is needed to make this approach more broadly applicable, e.g., to improve the classification performance, microbial signatures combined from early biofilms and saliva might provide practitioners with helpful information for advising habit changes to reduce caries risk.

## Methods

### Subjects

Twenty-seven healthy adults were enrolled in this study. Thirteen of them presented no cavitated caries lesions (NC group) and no signs of current clinical caries activity according to the Nyvad criteria for caries lesion activity^47^. Fourteen volunteers had by minimum three caries lesions reaching the dentine (CC group) and requiring restorative therapy. All participants were non-smokers and had no general diseases or diseases of the salivary glands. During a clinical oral examination, the following parameters were recorded: approximal plaque index (API) for oral hygiene, modified sulcus bleeding index (mSBI) for oral inflammation and, number of open caries lesions reaching the dentine for caries activity, caries indices DMFS (decayed, missing, filled surfaces) and ICDAS (International Caries Detection and Assessment System)^48^. In addition, information about age was registered and salivary flow rate was calculated in ml/min. The study design was reviewed and approved by the Ethics Committees of the Saarland (Sn52/05/2009) and Dresden (EK275092012) Universities. All experiments were performed in accordance with relevant guidelines and regulations. Written informed consent was obtained from all participants.

### *In-situ*-formation of oral biofilms

Enamel slabs (diameter 5 mm × 1 mm) were prepared from the labial surfaces of bovine incisors of BSE-negative 2-year old cattle. The surfaces of all samples were polished by wet grinding with abrasive paper (320–4000 grit). The smear layer on the slabs was removed by ultrasonication in 3% NaOCl for 3 min. Then, samples were washed twice in distilled water for 5 min and disinfected in 70% ethanol for 10 min under ultrasonication, respectively. Finally, the slabs were stored in fresh distilled water for 24 h before oral exposure^18,49^. Alginate impressions (Blueprint cremix®, Dentsply DeTrey, Konstanz, Germany) were taken from the upper jaws of all volunteers. Transparent custom made individual splints were fabricated as carriers of the bovine enamel specimens. Enamel slabs were fixed onto the splints in the left and right buccal position in the molar and premolar regions with polyvinyl siloxane impression material (Aquasil, Denstply De-Trey, Konstanz, Germany). The splints were exposed intraorally for 2, 4 and 8 h, respectively (Supplemental Fig. S6). Two slabs per time point were used per subject, one on the left side and one on the right side. Two hours before wearing the splints, the subjects brushed their teeth without toothpaste, flossing was optional. No meals or tooth brushing were allowed during exposure times. After intraoral exposure, specimens were rinsed for 10 s with sterile water. One ml of unstimulated saliva was obtained from each participant after 4 h by continuously collecting saliva in a sterile tube. No individuals with hypo- and hypersalivation were included in the study. All samples were stored at −20 °C until further processing.

### DNA preparation and next generation sequencing

DNA was extracted from biofilm samples and saliva samples using a commonly used DNA preparation kit (Qiagen DNA Blood and tissue kit, Qiagen, Hilden, G). Briefly, enamel slabs were placed in a 2 ml microcentrifuge tube and vortexed for 30 s at 2,500 rpm in 180 µl lysozyme-Triton TE-solution (20 mM Tris-Cl, pH 8.0, 2 mM sodium EDTA, 1.2% Triton® X-100, lysozyme 20 mg/ml) and stored at 37 °C for 30 min. Then, 20 µl of Proteinase K were added and the samples were thoroughly vortexed again for 30 s. After adding 200 µl Buffer AL and incubation at 56 °C for 60 min samples were placed on ice for 2 min. The next steps followed the bacterial and tissue protocol exactly. DNA was suspended in 20 µl of water and the DNA prepared from the two corresponding enamel plates was pooled. After preparation, DNA concentration was measured photometrically (Nanodrop 2000c, Thermo Fisher Scientific, Wilmington, DE, USA). PCRs of the 16S rRNA gene V1 and V2 variable regions were performed (5–25 ng template, 80 mM Tris-HCL, 20 mM (NH_4_)_2_SO_4_, 0.2% Tween-20, 2.5 mM MgCl_2_, 0.2 mM of each dNTP, 2.5 U HotFirePol (Solis BioDyne, Tartu, Est) using 200 pmol of each primer (forw16S_27F: 5′-agagtttgatcmtggctcag-3′, rev16S_338R: 5′-tgctgcctcccgtaggagt-3′) with Illumina universal adaptor sequences attached at the 5′-end. PCRs were performed in a thermocycler starting with 15 min at 95 °C followed by 33 cycles 95 °C for 1 min, 54 °C for 1 min, 72 °C for 1 min and a 5 min final extension at 72 °C. Amplicons were purified with Agencourt Ampure XP beads (BeckmanCoulter, Krefeld, Germany), diluted, pooled and sequenced (v3 chemistry: 2 × 300 bp paired-end) on the Illumina MiSeq (Illumina, San Diego, Ca, USA) following the manufacturer´s instructions aiming at 30,000 reads per sample.

### Sequencing control samples

For control purposes, enamel slabs without biofilms were fixed on acrylic splints and incubated at 37 °C for 2, 4 and 8 h in a sterile glass beaker under dry conditions. DNA was prepared from these specimens as described above. Additionally, DNA preparation was carried out with sterile water (PCR control) and a non-template control (NTC) were generated and sequenced.

### Sequence analysis

In a parallel study, children-derived CC and NC samples were generated and sequenced. Sequencing datasets from the present, adult samples and children samples of the parallel study were processed using LotuS, as described below. This joint processing was performed in order to generate common OTUs. OTUs uniquely present in the parallel samples were removed before further analysis in the present study. The sequencing reads in FASTQ-format were processed with LotuS version 1.47 including primer removal^50^. The default parameters of the sdm-MiSeq protocol were used, in particular, minSeqLength 170, minAvgQuality 27, TruncateSequenceLength 170, maxAccumulatedError 0.75. Operational taxonomic units (OTUs) were defined based on 97% sequence identity. In addition to the taxonomic assignments created by LotuS, the expanded Human Oral Microbiome Database (eHOMD^23,24^) was used to classify the OTUs. BLAST search (v. 2.2.23) was performed using the provided webserver (http://www.homd.org/?name = RNAblast&link = upload) in the database eHOMD 16S rRNA RefSeq version 15.1 with start at position 28 (parameters were set to default values). Top hits were extracted and saved including hit taxon, identity values and the number of mismatches. Relative OTU abundances were computed by dividing OTU read counts per sample by the respective total read count. OTUs present in any of the control samples (NTC, enamel, PCR) were discarded: OTUs detected in the NTC were discarded before computing the OTUs’ relative abundances, OTUs present in the other controls (PCR, enamel) were discarded after computing the relative abundances. PCR and enamel controls are considered as experiment-specific background. To account for this, we included these controls also during the bioinformatic steps and corrected for them following the computational analyses. Krona plots^51^ were created to provide an overview of the taxonomic composition of the dataset for all OTUs, for OTUs of 2 h, 4 h, and 8 h biofilm samples as well as from saliva samples. In addition, a Krona plot was prepared for the OTUs present in NTC, enamel and PCR controls. For the ordination analysis, the vegdist function and the Jaccard distance from the vegan R package, and the cmdscale function in the stats R package were used.

### Statistical analysis

All statistical analyses were performed using R^52^. Plots were mainly generated using ggplot2^53^. The biom-format output from LotuS, including the OTU relative abundance values, sample information, and taxonomy information, was used as input for phyloseq^54^. The Shannon Diversity index was used to estimate the alpha-diversity and the number of unique OTUs to estimate the richness. The two-tailed Wilcoxon-Mann-Whitney test (alpha = 0.05) was used to compare the diversity, the richness, and the relative abundance distributions of the individual OTUs between the cavitated-caries and no-cavitated-caries groups for each time point (2 h, 4 h, 8 h, saliva) after removal/correction of likely-contaminant OTUs. Raw p-values were adjusted using the Benjamini & Hochberg method for multiple comparisons^55^. Moreover, for each OTU, the area under the curve (AUC) was computed using the rocdemo.sca function from the made4 package^56^. The randomForest function from the randomForest package was used to build classification models in the case of OTU signatures, i.e., OTU sets, for the individual time points (2 h, 4 h, 8 h, saliva)^57^. Furthermore, the Spearman correlation coefficient was computed using the core function in R.

### Ethics approval and consent to participate

The study design was reviewed and approved by the Ethics Committees of the Saarland (Sn52/05/2009) and Dresden (EK275092012) Universities. All experiments were performed in accordance with relevant guidelines and regulations. Written informed consent was obtained from all participants.

## Electronic supplementary material

Supplementary Figures 2 and 3

Supplementary Information

Supplementary Dataset

## Data Availability

The datasets generated during and/or analyzed during the current study are available from the corresponding author on reasonable request basis.
