# Supplementary material for: Comparison of initial oral microbiomes of young adults with and without cavitated dentin caries lesions using an in situ biofilm model
Source: Sci Rep. 2018 Sep 18;8:14010. doi: 10.1038/s41598-018-32361-x (PMC6143549; doi:10.1038/s41598-018-32361-x)
Supplement: Supplementary file 1 — Supplementary Figures 2 and 3 [file 41598_2018_32361_MOESM1_ESM.zip › Supplementary_Figure_2a.html]

Javascript must be enabled to view this page.

magnitude
magnitudeUnassigned

main\_otus\_\_all

1875
3

359
1872

10

10

2

2

1

1

8

7

7

1

1

8

2

2

2

2

1

1

1

1

5

5

5

5

5

5

5

5

5

1

1

1

1

1

4

4

4

4

288
56

56

56
1

21
37

2

2

2

4

1

2

1

2

3
16

1

7

3

2

2

2

75

75
1

2

2

10
72

1

35

9

1

16

3

3

3

3

81

6
81

2

2

1
13

3

5

4

60
4

5

1

50

17

17

1
16

1

2

1

2

2

2

5

1

55

55

7
55

19
2

17

12
29

2

11

4

40

40

40

40

40

287
15

3

3

3

1

1

1

54

54

54
10

2

17

2

1

8

4

3

5

1

1

3
102

4
27

10
1

7

2

1

1

8

8

3

1

2

1

1

72
3

5

4

1

33

31

2

14

12

1

1

3

1

2

12

12

2

1

1

113
1

26
112

12
1

3

1

5

1

1

2

5
32

7

3

7

1

1

2

1

3

1

1

8

3

5

11

2

4

1

1

3

10
3

7

9
4

1

1

1

2

2

2

2
6

3

3

3

3

1

1

1

1

3

3

3

3

3

1

1

1

1

1

4

4

4

4

4

1
185

3
184

7
6

1

1

5

5

3

1

1

3

3

3

3

3

1

1

1

4
3

1

1

24
159

22

22

12
1

1

3

6

1

7

7

1

1

6

5

1

2

2

4

3

1

4

1

3

5

1

1

1

1

1

1

1

20
8

10

1

1

2

2

1
36

34

1

6
12

1

1

3

1

1

1

12

10

10

10

3

3

1

3

2
1

1

1

1

75
529

28
126

1

1

1

1

1

1

3

3

3

1

1

1

5

2
5

1

1

1

26
1

1
13

2

1

8

1

1
12

1

2

8

2

1
2

1

14
1

2

1

1

11
6

1

1

1

2

13

13
9

1

3

1

1

1

31

27

27

4

1

3

28
6

1

1

1

6
14

1

6
7

1

6

6

6

1

1

1

8

8

1

1

7

2

5

100
23

3

1
3

1

1

2

2

2

1

1

1

1

1

1

18

18
7

6

1

1

3

13

2
13

1

2

3

2

1

1

1

7
39

2
4

1

1

5

1

1

1

1

1

5
17

1

2

1

1

2

2

1

1

1

2

1

1

4

1

3

192
45

19

19
10

1

1

4

1

2

57
20

1

2
8

2

3

1

6
1

5

1
5

2

2

1

1

4

3

1

3
2

1

1

8

6

2

40

40
13

5

1

2

13

1

5

18
3

4

1

1

2

7
11

1

1

1

1

13
1

1
12

6

2

3

2

2

2

2
1

1

23

2

2

2

2

1
4

3

3

3

1

1

1

1

3

3

3

3

3

3

3
1

2

7
1

6

6

6

3

3

3

3

30

30

30

30

30

20

20

20

18

18

2

2
