# Supplementary material for: Comparison of initial oral microbiomes of young adults with and without cavitated dentin caries lesions using an in situ biofilm model
Source: Sci Rep. 2018 Sep 18;8:14010. doi: 10.1038/s41598-018-32361-x (PMC6143549; doi:10.1038/s41598-018-32361-x)
Supplement: Supplementary file 1 — Supplementary Figures 2 and 3 [file 41598_2018_32361_MOESM1_ESM.zip › Supplementary_Figure_2b.html]

Javascript must be enabled to view this page.

magnitude
magnitudeUnassigned

ctrl\_otus

506
1

505
36

1

1

1

1

1

183
6

43
2

5

5

5

3
30

3

2

1

7
1

1

1

1

2

1

4

1

1

1

1

2
12

3

2

1

3

1

1

2

2

1

1

2

2

1

1

2

1
2

1

69
5

10

10
5

1

4

24

14

9

3

1

1

10
1

1

7

1

6

2

1

1

1

1

3

3

1

1

1

3

1
3

2

1

1

1

8

7
2

1

1

3

1

1

1

1

1

4

2
4

1

1

6

1

1

4

1

3

1

1

58
3

4

1

3

1

1

1

14

13
2

2

9

1

5

2
5

2

1

6

6
1

1

3

1

26
3

1

1

3

12

12

1

1

2
1

1

4
1

1

2

3
7

1

1

1

3

2

1

1

1

1

1

6

6

6

2

1

1

1
4

3

81

81

2

1
2

1

1

1

1

12
76

8

8

1

1

9
6

2

1

1

1

9

1

6

1

1

1

1

1

1

11

4

2

1

4

12

1

11

1

1

2

1

1

4

3

1

1

1

1

1

2

2

1

1

1

1

1

1

5

5

5

5

5

2

1

1

1

1

1

1

1

1

1

1

1

1

1

6
62

15

1
15

4

1

3

2
10

8

5

5

3

2

1

1

11

11

11
2

1

1

1

2

4

25

25
2

7
22

2

3

3

5

2

1

1

12

12

12

12

12

6

5

5

5

2

3

1

2

2

2

2

2

1
102

74

53

3

3

2
6

2

1

1

35

35

4

1

3

5

5

21
2

1

1

6

5

1

6

2

2

2

6

6

1

1

1

1

8

8

1
8

3

1

3

18

18
2

5
3

1

1

1

1

7

2

1

1

3

3
2

1

3

3

2

1

1

1

1

1

1

1

1

1

1

1
