# Supplementary material for: Comparison of initial oral microbiomes of young adults with and without cavitated dentin caries lesions using an in situ biofilm model
Source: Sci Rep. 2018 Sep 18;8:14010. doi: 10.1038/s41598-018-32361-x (PMC6143549; doi:10.1038/s41598-018-32361-x)
Supplement: Supplementary file 1 — Supplementary Figures 2 and 3 [file 41598_2018_32361_MOESM1_ESM.zip › Supplementary_Figure_3a.html]

Javascript must be enabled to view this page.

magnitude
magnitudeUnassigned

main\_otus\_\_2h

1090
2

195
1088

3

3

3

3

3

5

3

3

3

3

2

2

2

2

20

20

20

20

20

10

3

3

3

3

1

1

1

1

1

1

1

1

2

2

2

2

1

1

1

1

1
2

1

1

1

2
1

1

1

1

1

1

1

1

1

1

1

1

1

1

3
135

2

2

2

1

1

62
1

14
61

7

2

1

3

1

2

3

1

1

1

1
3

1

1

2
9

7

20
3

4

1

1

1

2

6

1

1

3

2

1

2
51

14
2

5

4

1

2

1

1

4

4

1

1

1
35

2

2

20

18

2

4

4

2

1

1

5

1

3

1

1

1

17

17

17
4

1

1

2

5

2

1

1

353
51

18
85

9

7
3

2

1

1

2

1

1

1

1

1

8

8
5

2

1

1

1

1

2

2

2

23

3

1

2

20

20

1

1

1

1
15

1
8

4

2

1

6
1

2

1

2

2

1
2

1

1

1

1

4

4
1

1

1

1

14
66

8

8
1

1

2

3

1

2

2

2

1

1

1

31
6

2
3

1

3
14

1

1

1

2

1

2

1

1

1

4

1

1

1

1

3

1

2

1

1

7

7

2

1

1

1

1

1

3

1
3

1

1

7

7

1

1

6

1

5

127
26

10
36

4

3

1

1

1

1

1
7

2

3

1

3

3

1

1
4

3

4
1

2

1

1

11

5
11

1

2

3

10

10

3

5

2

34

12
34

1

4

2

4

1

10

1
10

3

1

2

6
4

1

1

17
3

2

2

2

1

1

1

1

1

1

10
3

1

6
5

1

2

2

2

2

2

8

8

8

2

2

6

6

29

29

29
3

12
1

11

14
7

1

6

8

7

7

7

2

3

2

1

3

3

3

3

1
119

118

3

3

3

104
16

14

14

3

1

2

1
23

22

7

7

1

1

2

1

1

1

1

4

1

3

5

2

2

1

4
9

1

3

1

3

1

1

1

2

2

4
12

8

2

2

2
3

1

1

4
3

1

1

4

4

1

2

1

1

1

1

1

1

20

20

20

20

20

7

7

5

4

4

1

1

2

2

1

1

166
33

10

10

1
9

1

1

3

1

1

1

1

33

33
2

6

2

2

2

25
2

19

4

39

39
1

26
14

1

1

1

4

2

2

1

1

1

11
1

1

2

1

6

48

48

7
46

1

24

5

9

2

2

3

3

3

3
