# Supplementary material for: Comparison of initial oral microbiomes of young adults with and without cavitated dentin caries lesions using an in situ biofilm model
Source: Sci Rep. 2018 Sep 18;8:14010. doi: 10.1038/s41598-018-32361-x (PMC6143549; doi:10.1038/s41598-018-32361-x)
Supplement: Supplementary file 1 — Supplementary Figures 2 and 3 [file 41598_2018_32361_MOESM1_ESM.zip › Supplementary_Figure_3b.html]

Javascript must be enabled to view this page.

magnitude
magnitudeUnassigned

main\_otus\_\_4h

912
2

910
155

1

1

1

1

1

2

2

2

2

27
141

9

9

9
1

2

1

2

2

1

24

24
1

2
16

12

2

1

1

6
1

1

2

2

47

47

2

2

6
45

8

1

9

21

34

34

1

1

2
9

2

5

12
24

2

2

1

2

1

1

3

6

1

1

1

1

5

5

5

2

1

2

4

4

4

4

4

4

4

4

4

4

2

2

2

2

2

14

14

14

14

14

30

30

3
30

12

12

8
15

1

4

2

1

1

1

1

1

2
3

1

1

1

1

3

1

1

1

1

2

2

2

2

15

3

3

3
1

2

2

2

2

2

1
3

2

2

2

1

1

1

1

1

1

1

1

4

4

4

4

1

1

1

1

17

17

17

17

17

308
35

5

5

4

1

3

1

1

11
69

16

1
8

1

6

8

1

1

1

5

1
11

8
5

1

1

1

2

1

1

3

3

3

1

1

1

1

1

16

15

15

1

1

7

7
5

1

1

3

1
3

1

1

13
63

5
26

3

3

2

1

1

10
4

1

1

1

1

1

1

2

1

1

4

1

1

1

1

1

1

1

1

1

1

2

2

1

1

10

2
10

2

1

3

1

1

9

2
9

1

4

2

1

1

1

10
2

3
5

2

3

3

3

26
126

10
2

2

1

1

4
6

1

1

10

10
4

1

1

1

1

2

9

1
9

1

1

6

30

8
30

4

10

1

2

5

41
12

1

1

1

1
6

3

2

1
5

4

1
2

1

4

1

3

1

5

1

4

4
1

2

1

3
109

13

13

13

1

1

7

1

1

1

1

1
53

13
1

5

5

1
4

3

2

2

1

1

39

1

1

6

4

1

1

25

1

24

4

4

1

1

2

2

1

1

1

1

39

2
39

2
5

3

1

5
1

1

1

2

2
5

2

1

13

4

6

1

1

1

5

2

3

3

1

2

94
1

3
93

1

1

1

1

3

82
8

1

1

4
3

1

24

1

23

5

5

2

2

16
6

1

9

2

2

1

1

7
1

1

2

3

12

12

1

1

1

2

2

1

1

1

1

1

1

1
