# Supplementary material for: Comparison of initial oral microbiomes of young adults with and without cavitated dentin caries lesions using an in situ biofilm model
Source: Sci Rep. 2018 Sep 18;8:14010. doi: 10.1038/s41598-018-32361-x (PMC6143549; doi:10.1038/s41598-018-32361-x)
Supplement: Supplementary file 1 — Supplementary Figures 2 and 3 [file 41598_2018_32361_MOESM1_ESM.zip › Supplementary_Figure_3c.html]

Javascript must be enabled to view this page.

magnitude
magnitudeUnassigned

main\_otus\_\_8h

2
874

109
872

2

2

2

2

1

1

1

1

1

1

1

13

13

13

11

11

2

2

1

1

1

1

1

15

15

15

15

15

31
258

8
53

2

2

1

1

4
24

1

1

5

1

1

1

1

1

2
12

1

1

1

1

1

1

1

2

1

2

2

1

1

1

12

12
3

1

5

2

1

6

1
6

1

2

1

1

3
14

6

6

6

5
3

2

5

5

5

5

100
18

9

9
4

4

1

23

6
23

3

5

2

1

6

1
9

8

5

1

2

1
8

6
4

1

1

1

1

10
33

3
1

1

1

5

1

2

2

1

1

1

1

4
1

3

1

3

1

2

5

1

4

9
55

1

1

1

1

1

1

8

1

1

7

7

13

6
1

4

1

1
7

6

10

10
7

2

1

1

1

1

2

2

2

1
8

2

1

1

3
5

1

1

2

2

11
134

28

28

12
20

2

1

1

2

1

1

1

1

7
1

2

3

1

42

42
1

1

1

33
3

1

4

25

7

3

3

1

43

1
43

2

2

8
40

1

3

15

13

10

10

10
1

1

4

1

2

1

2

2

2

2

2

1

1

1

1

1

2

1

1

1

1

1

1

1

1

17

17

17

17

17

8

2

2

2

2

1

1

1

1

2
1

1

1

1

3
1

2

2

2

39

39

39
4

15

15

20
9

10

1

1

1

1

1

1

98
1

97

9
89

26

1

25

5
12

1

1

5

3

3

3

1

2

1

1

4

4

5
4

1

2

1

1

2

2

1

1

1

1

17

17

3

3

2

4

4

2

1

1

1

1

1

1

10
170

3

3

3

1

1

1

33

33

33
5

12

1

6

4

4

1

69
3

2
14

4

3

1

1

1

6

6

1

1

52
1

4

3

1

7

7

2

1

1

3

1

2

29

28

1

6

6

55

55
10

7

1

3

1

2

1

1

5

5

1

4
21

2

2

1

6

5

1

6

1

4

1

4
2

1

1

1

1

1

1

1
