# Supplementary material for: Comparison of initial oral microbiomes of young adults with and without cavitated dentin caries lesions using an in situ biofilm model
Source: Sci Rep. 2018 Sep 18;8:14010. doi: 10.1038/s41598-018-32361-x (PMC6143549; doi:10.1038/s41598-018-32361-x)
Supplement: Supplementary file 1 — Supplementary Figures 2 and 3 [file 41598_2018_32361_MOESM1_ESM.zip › Supplementary_Figure_3d.html]

Javascript must be enabled to view this page.

magnitude
magnitudeUnassigned

main\_otus\_\_Saliva

499

499
36

57

57

5

5

1

1

3

3

3

1

1

1

5
49

3

3

7

7

31

30

1

2
3

1

95
9

22

22

22
6

16

64

64
4

53
4

5

1

43

7

4

3

17

17

17

17

17

50

50

50
6

26
12

2

10

2

18
1

17

23

23

23

23

23

175
13

55

55
13

5

1

4

5

1

1

3

5

5

3
25

7

1

3

1

7

3

2

2

61
3

8

1

1

7

7

2
50

7

7

1

1

24

23

1

12

12

4

4

2

2

2

1

1

44

44

44
6

2

4

2

15

5

3

7

1

1

1

1

1

1

1

1

1

1

44
3

14
1

10

10
6

3

1

3

3

3

2

1

1

1

1

1

1

5

5

5

5

1
20

1

1

1

1
3

2
1

1

15

5
15

3

5

1

1
