# Supplementary material for: Comparison of initial oral microbiomes of young adults with and without cavitated dentin caries lesions using an in situ biofilm model
Source: Sci Rep. 2018 Sep 18;8:14010. doi: 10.1038/s41598-018-32361-x (PMC6143549; doi:10.1038/s41598-018-32361-x)
Supplement: Supplementary file 2 — Supplementary Information [file 41598_2018_32361_MOESM2_ESM.docx]

**Supplementary Material for**

**Comparison of initial oral microbiomes of young adults with and without cavitated dentin caries lesions using an *in situ* biofilm model**

Stefan Rupf^1*^, Cedric C. Laczny^2^, Valentina Galata^2^, Christina Backes^2^, Andreas Keller^2^, Natalia Umanskaya^1^, Arzu Erol^1^, Sascha Tierling^3^, Christina Lo Porto^3^, Jörn Walter^3^, Jasmin Kirsch^4^, Matthias Hannig^1^, Christian Hannig^4^

^1^ Clinic of Operative Dentistry, Periodontology and Preventive Dentistry, Saarland University Medical Center, Homburg, Germany,

^2^ Chair for Clinical Bioinformatics, Saarland University, Germany,

^3^ Faculty of Natural Sciences and Technology, Department of Genetics/Epigenetics, University of Saarland, Germany,

^4^ Policlinic of Operative and Pediatric Dentistry, Medical Faculty Carl Gustav Carus, TU Dresden, Germany

[stefan.rupf@uks.eu](mailto:stefan.rupf@uks.eu), *corresponding author

**
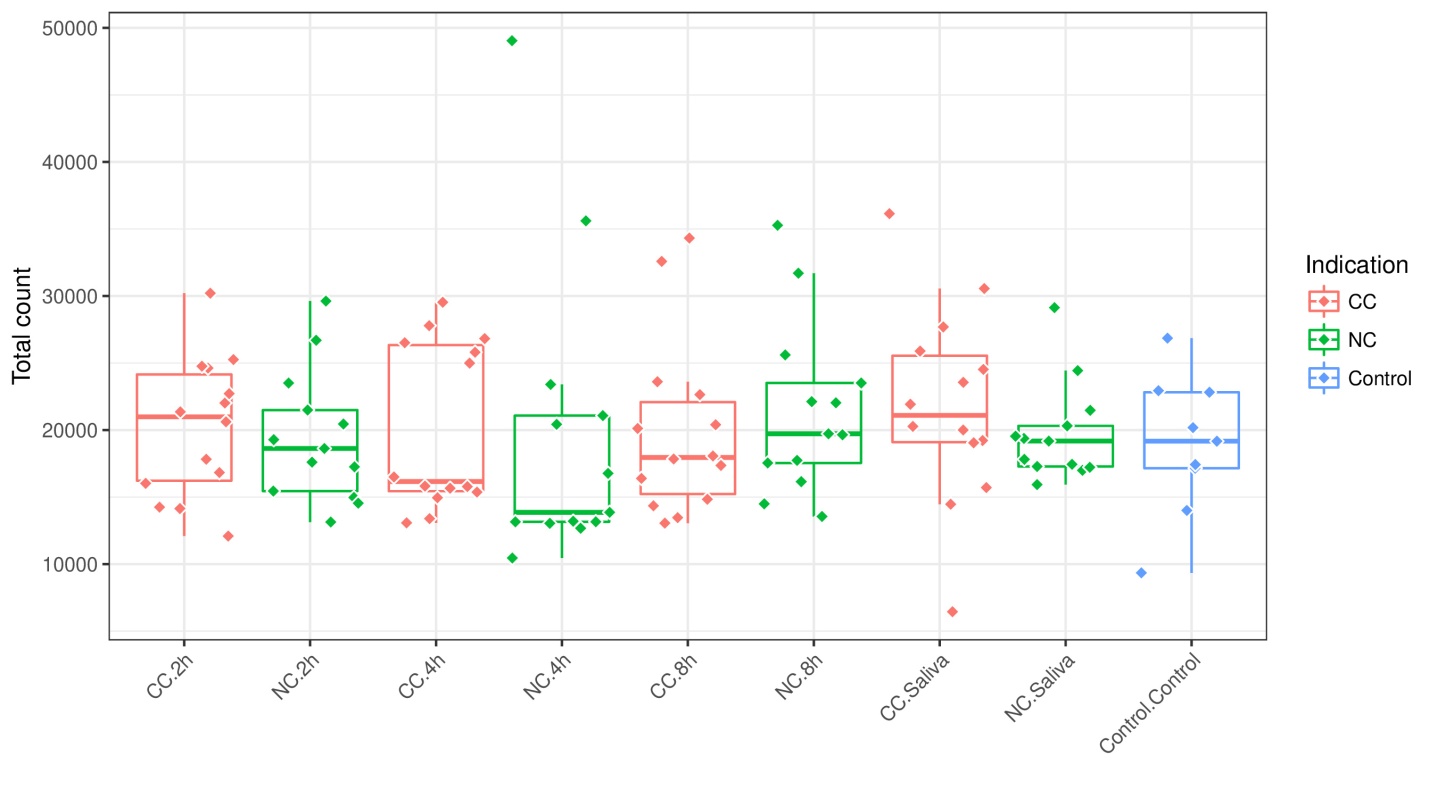
**

**Figure S1**: Total OTU count per sample and sample groups. The samples were grouped by type (2 h, 4 h and 8 h biofilm samples, and saliva samples) and indication (CC, NC, and control). The boxplots show the distribution of total OTU counts per sample within the respective groups. The dots demonstrate the total OTU counts per sample. The colors highlight the sample indication as CC, NC, or control.

**Figure S2 (please open according HTML-files)**: Krona plots showing the taxonomic composition of sets of OTUs w.r.t. taxonomic lineages assigned by LotuS. The depicted OTU sets comprise all OTUs without OTUs present in at least one control probe (a) and OTU present in at least one control probe (b).

[**Supplementary_Figures 2_3.zip**](file:///C:\Users\rupf_s\AppData\Local\Microsoft\Windows\Temporary%20Internet%20Files\Content.IE5\H8SN7K12\Supplementary_Figures%202_3.zip)

**Figure S3 (please open according HTML-files)**: Krona plots showing the taxonomic composition of sets of OTUs w.r.t. taxonomic lineages assigned by LotuS. The depicted OTU sets comprise all OTUs present in 2h- (a), 4h- (b), 8h-bioflim samples (c), and in saliva (d), respectively. OTUs present in any control probe were removed.

[Supplementary_Figures 2_3.zip](file:///C:\Users\rupf_s\AppData\Local\Microsoft\Windows\Temporary%20Internet%20Files\Content.IE5\H8SN7K12\Supplementary_Figures%202_3.zip)


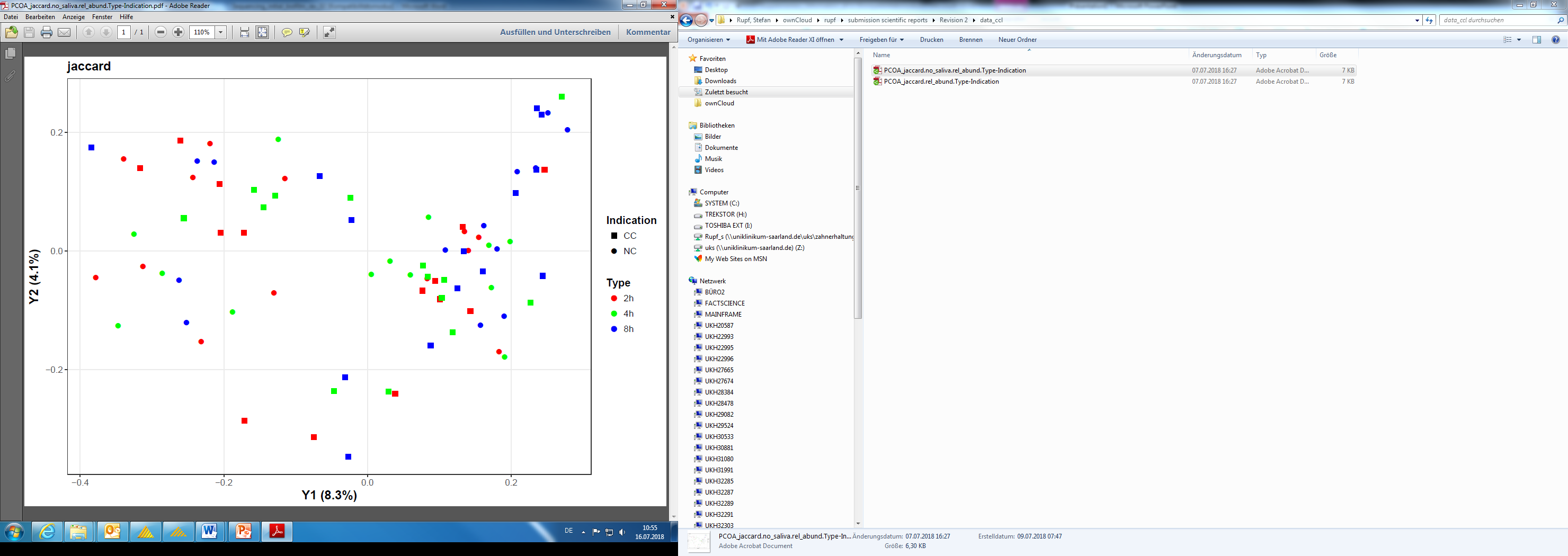


**Figure S4**: PCoA-based ordination of the Jaccard distance matrix. Colors reflect the sample type (2 h, 4 h, and 8 h) and shapes reflect the CC and NC groups. The percentage of variation explained is included in the axis labels.


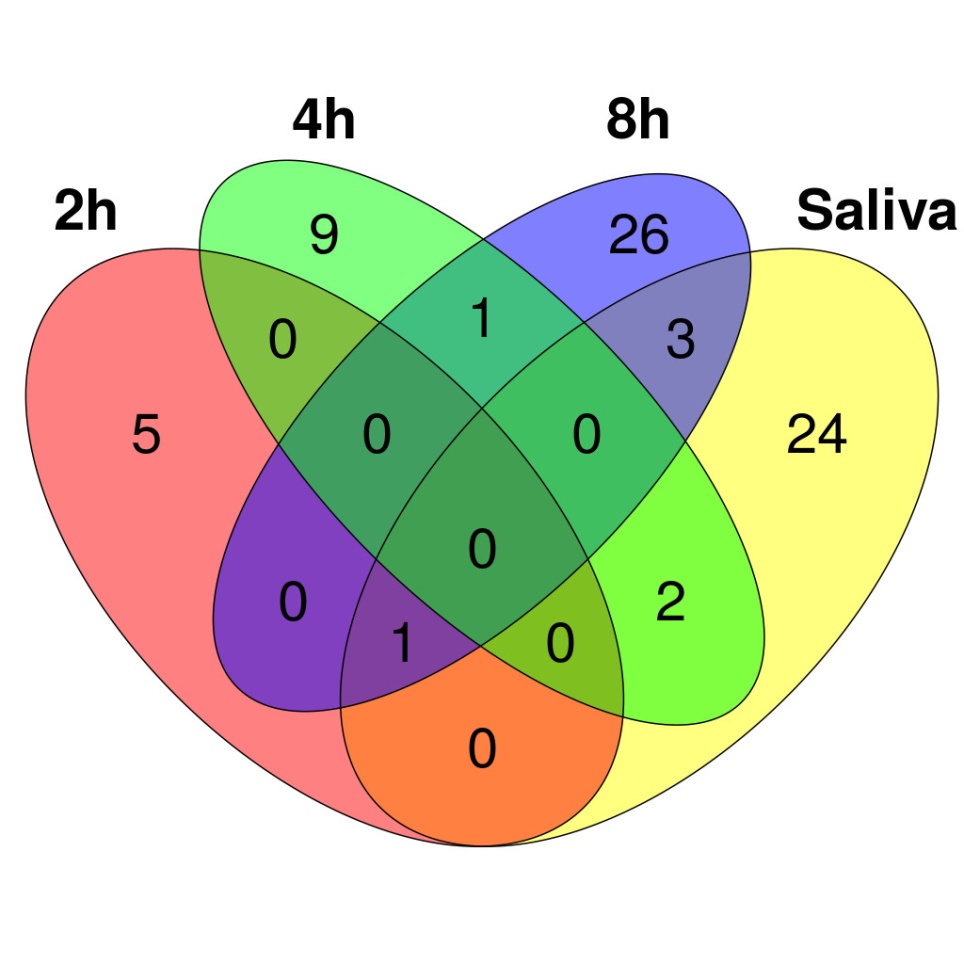


**Figure S5**: Number of and overlaps between statistically significantly differentially abundant OTUs per time point for the comparison of samples in CC and NC groups.


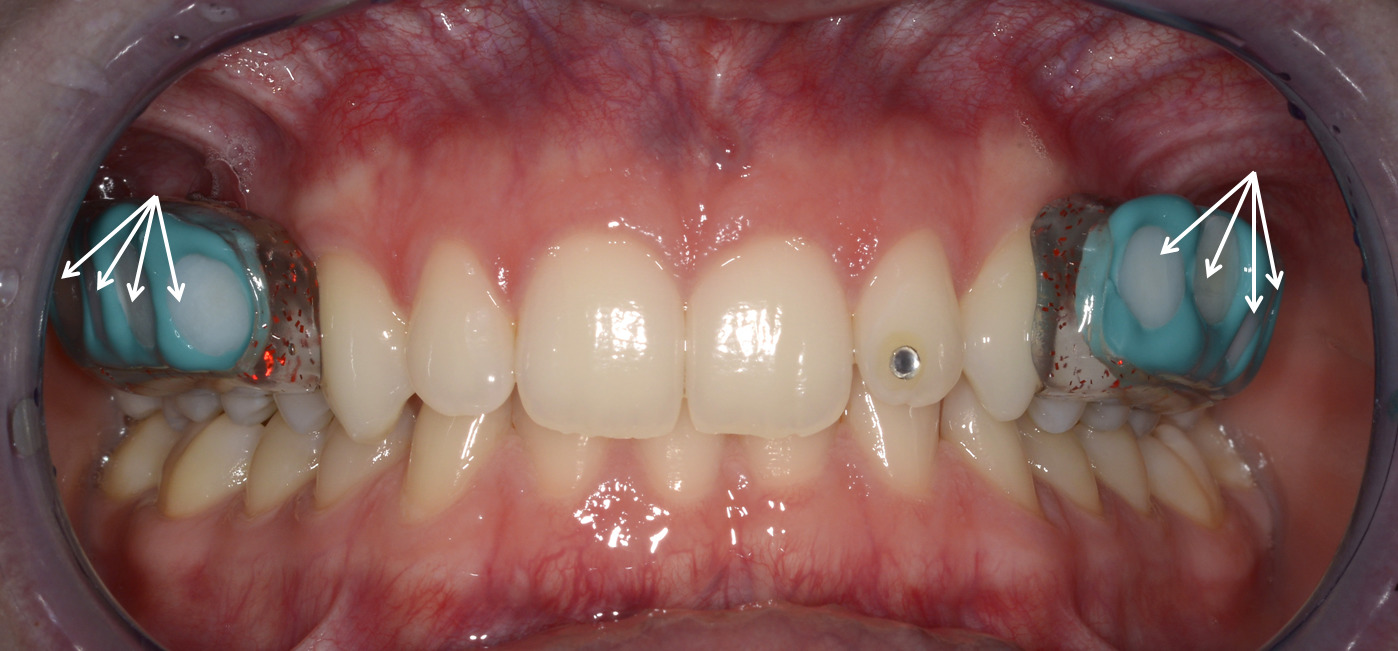


**Figure S6**: Individually removable acrylic splints, placed in the upper jaw. The specimens (arrows) are fixed with silicone in the left and right buccal regions of the upper premolars to the first molar.

**Supplementary Tables**

**Supplementary Tables 1**: Supplemental table 1: (a) Clinical data overview of study participants with cavitated dentin caries (CC) and without cavitated caries (NC). Mean and standard deviation are given. DMFS: decayed, missing, filled surfaces, DS: decayed surfaces, ICDAS: International Caries Detection and Assessment System; mean number of surfaces for cavitated caries/subject: 132.8 (occlusal (occ): 17, oral/vestibular (o/v): 58, mesial/distal (m/d): 58), for no cavitated caries/subject: 132.4 (occlusal: 16.9, oral/vestibular: 57.8, mesial/distal: 57.8). Percentages are given for scores 0, scores 1/2: first or distinct visual changes in enamel, scores 3/4: localized enamel breakdown or underlaying dark shadow from dentin, scores 5/6: distinct or extensive distinct cavity in dentin, age, gender, mSBI: modified sulcus bleeding index, API: approximal plaque index.

|  | **Cavitated caries** | | | **No cavitated caries** | | |
| --- | --- | --- | --- | --- | --- | --- |
| **DMFS** | 22.7 ± 12.1 | | | 1.5 ± 1.8 | | |
| **DS** | 4.4 ± 1.8 | | | 0 | | |
| **ICDAS % surfaces** | occ | o/v | m/d | occ | o/v | m/d |
| **ICDAS score 0** | 39,30% | 79,60% | 76,70% | 31,80% | 92,30% | 93,60% |
| **ICDAS scores1/2** | 24.4 ± 9.2% | 8.8 ± 2.2% | 7.6 ± 1.3% | 34.1 ± 1.3% | 3.9 ± 3.1% | 3.2 ± 2.4% |
| **ICDAS scores ¾** | 5.05 ± 1.2% | 1.2 ± 1.5% | 3.1 ± 0.1% | 0,00% | 0,00% | 0,00% |
| **ICDAS scores 5/6** | 0.9 ± 0.4% | 0.1 ± 0.1% | 0.9 ± 1.3% | 0,00% | 0,00% | 0,00% |
| **Age (mean, years)** | 24,6 | | | 25,2 | | |
| **Age (range, years)** | 20 – 30 | | | 19 – 33 | | |
| **Ratio female : male** | 43 % : 57 % | | | 69 % : 31 % | | |
| **mSBI** | 44.6 ± 23.8 % | | | 35.2 ± 24.2 % | | |
| **API** | 70.8 ± 27.7 % | | | 61.6 ± 29.4 % | | |
| **Salivary flow rate** | > 0.25 ml/min, < 1.0 ml / min | | | | | |

(b) Clinical data listed per subject including DMFS, DS, gender, age, mSBI, API. Note: Subjects with active enamel caries lesions (white spots) were excluded from group “no cavitated caries”. The visible changes in the enamel were brown discolorations, the surrounding enamel appeared glossy.

| **CC** | **DMFS** | **DS** | **Gender** | **Age** | **mSBI** | **API** |
| --- | --- | --- | --- | --- | --- | --- |
| 1 | 24 | 3 | M | 28 | 78 | 100 |
| 2 | 20 | 3 | F | 25 | 67 | 96 |
| 3 | 14 | 8 | M | 25 | 33 | 80 |
| 4 | 18 | 4 | M | 23 | 36 | 96 |
| 5 | 54 | 5 | F | 30 | 86 | 100 |
| 6 | 26 | 3 | M | 24 | 50 | 65 |
| 7 | 19 | 4 | F | 23 | 39 | 93 |
| 8 | 11 | 4 | F | 23 | 43 | 79 |
| 9 | 26 | 7 | M | 22 | 75 | 75 |
| 10 | 15 | 3 | F | 21 | 27 | 77 |
| 11 | 22 | 8 | M | 20 | 43 | 47 |
| 12 | 29 | 4 | M | 25 | 22 | 23 |
| 13 | 24 | 3 | M | 30 | 12 | 25 |
| 14 | 16 | 3 | F | 20 | 14 | 35 |
| **NC** | **DMFS** | **DS** | **Gender** | **age** | **mSBI** | **API** |
| 1 | 2 | 0 | F | 33 | 46 | 75 |
| 2 | 0 | 0 | F | 25 | 17 | 71 |
| 3 | 0 | 0 | F | 22 | 42 | 83 |
| 4 | 0 | 0 | F | 22 | 39 | 64 |
| 5 | 5 | 0 | M | 23 | 58 | 71 |
| 6 | 3 | 0 | F | 27 | 71 | 96 |
| 7 | 1 | 0 | F | 30 | 42 | 75 |
| 8 | 0 | 0 | F | 27 | 73 | 82 |
| 9 | 3 | 0 | M | 26 | 25 | 86 |
| 10 | 0 | 0 | M | 29 | 0 | 11 |
| 11 | 0 | 0 | M | 19 | 0 | 8 |
| 12 | 4 | 0 | F | 24 | 36 | 59 |
| 13 | 2 | 0 | F | 20 | 9 | 20 |

(c) Sample information table including sample ID (column “UniqueSeqID”), grouping into CC/NC/controls (column “Indication”, “1” = CC, “2” = NC, “Kontrolle Schmelz” = enamel control, “Kontrolle-PCR” = PCR control, “Kontrolle-NTC” = NTC control), grouping into 2/4/8 h biosamples, saliva and controls (column “Type”), and subject ID (column “Individual”).

| **UniqueSeqID** | **Indication** | **Type** | **Subject ID** |  |
| --- | --- | --- | --- | --- |
| 140929-ad1_001 | 1 | 2h | 1 |  |
| 140929-ad1_003 | 1 | 2h | 2 |  |
| 140929-ad1_008 | 2 | 2h | 1 |  |
| 140929-ad1_009 | 2 | 2h | 2 |  |
| 140929-ad1_010 | 2 | 2h | 3 |  |
| 140929-ad1_011 | 2 | 2h | 4 |  |
| 140929-ad1_013 | 1 | 2h | 4 |  |
| 140929-ad1_014 | 2 | 2h | 5 |  |
| 140929-ad1_015 | 2 | 2h | 6 |  |
| 140929-ad1_016 | 2 | 2h | 7 |  |
| 140929-ad1_018 | 1 | 2h | 5 |  |
| 140929-ad1_019 | 1 | 2h | 6 |  |
| 140929-ad1_020 | 2 | 2h | 8 |  |
| 140929-ad1_021 | 1 | 2h | 7 |  |
| 140929-ad1_022 | 1 | 2h | 8 |  |
| 140929-ad1_023 | 2 | 2h | 9 |  |
| 140929-ad1_025 | 2 | 2h | 10 |  |
| 140929-ad1_027 | 1 | 2h | 11 |  |
| 140929-ad1_028 | 2 | 2h | 11 |  |
| 140929-ad1_034 | 2 | 2h | 12 |  |
| 140929-ad1_035 | 1 | 2h | 12 |  |
| 140929-ad1_036 | 2 | 2h | 13 |  |
| 140929-ad1_037 | 1 | 2h | 13 |  |
| 140929-ad1_038 | 1 | 2h | 14 |  |
| 140929-ad1_039 | 1 | 4h | 1 |  |
| 140929-ad1_040 | 1 | 4h | 2 |  |
| 140929-ad1_041 | 1 | 4h | 3 |  |
| 140929-ad1_042 | 2 | 4h | 1 |  |
| 140929-ad1_043 | 2 | 4h | 2 |  |
| 140929-ad1_044 | 2 | 4h | 3 |  |
| 140929-ad1_045 | 2 | 4h | 4 |  |
| 140929-ad1_046 | 1 | 4h | 4 |  |
| 140929-ad1_047 | 2 | 4h | 5 |  |
| 140929-ad1_048 | 2 | 4h | 6 |  |
| 140929-ad2_001 | 2 | 4h | 7 |  |
| 140929-ad2_003 | 1 | 4h | 5 |  |
| 140929-ad2_008 | 2 | 4h | 8 |  |
| 140929-ad2_009 | 1 | 4h | 6 |  |
| 140929-ad2_010 | 1 | 4h | 7 |  |
| 140929-ad2_011 | 2 | 4h | 9 |  |
| 140929-ad2_013 | 2 | 4h | 10 |  |
| 140929-ad2_014 | 1 | 4h | 11 |  |
| 140929-ad2_015 | 2 | 4h | 11 |  |
| 140929-ad2_022 | 2 | 4h | 12 |  |
| 140929-ad2_023 | 1 | 4h | 12 |  |
| 140929-ad2_025 | 2 | 4h | 13 |  |
| 140929-ad2_027 | 1 | 4h | 13 |  |
| 140929-ad2_028 | 1 | 4h | 14 |  |
| 140929-ad2_044 | 2 | 8h | 10 |  |
| 140929-ad2_045 | 1 | 8h | 11 |  |
| 140929-ad2_046 | 2 | 8h | 11 |  |
| 140929-ad3_018 | 2 | 8h | 12 |  |
| 140929-ad3_019 | 1 | 8h | 12 |  |
| 140929-ad3_020 | 2 | 8h | 13 |  |
| 140929-ad3_021 | 1 | 8h | 13 |  |
| 140929-ad3_022 | 1 | 8h | 14 |  |
| 140929-ad3_023 | Kontrolle-Schmelz | Kontrolle-Schmelz |  |  |
| 140929-ad3_025 | Kontrolle-Schmelz | Kontrolle-Schmelz |  |  |
| 140929-ad3_027 | Kontrolle-Schmelz | Kontrolle-Schmelz |  |  |
| 140929-ad3_028 | Kontrolle-Schmelz | Kontrolle-Schmelz |  |  |
| 140929-ad3_029 | 1 | 2h | 3 |  |
| 140929-ad3_030 | 1 | 4h | 6 |  |
| 141128-ad1_038 | 1 | 2h | 9 |  |
| 141128-ad1_039 | 1 | 4h | 9 |  |
| 141128-ad1_040 | 1 | 2h | 10 |  |
| 141128-ad1_041 | 1 | 4h | 10 |  |
| 141128-ad1_042 | 1 | 8h | 10 |  |
| 141128-ad2_001 | 1 | 8h | 1 |  |
| 141128-ad2_003 | 1 | 8h | 2 |  |
| 141128-ad2_008 | 1 | 8h | 3 |  |
| 141128-ad2_009 | 2 | 8h | 1 |  |
| 141128-ad2_010 | 2 | 8h | 2 |  |
| 141128-ad2_011 | 2 | 8h | 3 |  |
| 141128-ad2_013 | 2 | 8h | 4 |  |
| 141128-ad2_014 | 1 | 8h | 4 |  |
| 141128-ad2_015 | 2 | 8h | 5 |  |
| 141128-ad2_016 | 2 | 8h | 6 |  |
| 141128-ad2_018 | 2 | 8h | 7 |  |
| 141128-ad2_019 | 1 | 8h | 5 |  |
| 141128-ad2_020 | 1 | 8h | 6 |  |
| 141128-ad2_021 | 2 | 8h | 8 |  |
| 141128-ad2_022 | 1 | 8h | 7 |  |
| 141128-ad2_023 | 1 | 8h | 8 |  |
| 141128-ad2_025 | 2 | 8h | 9 |  |
| 141128-ad2_027 | 1 | 8h | 9 |  |
| 141128-ad2_042 | 2 | Saliva | 10 |  |
| 141128-ad2_043 | 1 | Saliva | 11 |  |
| 141128-ad2_044 | 2 | Saliva | 11 |  |
| 141128-ad3_003 | 2 | Saliva | 12 |  |
| 141128-ad3_008 | 1 | Saliva | 12 |  |
| 141128-ad3_009 | 2 | Saliva | 13 |  |
| 141128-ad3_010 | 1 | Saliva | 14 |  |
| 141128-ad3_011 | 1 | Saliva | 13 |  |
| 141128-ad3_013 | 1 | Saliva | 1 |  |
| 141128-ad3_014 | 1 | Saliva | 2 |  |
| 141128-ad3_015 | 1 | Saliva | 3 |  |
| 141128-ad3_016 | 2 | Saliva | 1 |  |
| 141128-ad3_018 | 2 | Saliva | 2 |  |
| 141128-ad3_019 | 2 | Saliva | 3 |  |
| 141128-ad3_020 | 2 | Saliva | 4 |  |
| 141128-ad3_021 | 1 | Saliva | 4 |  |
| 141128-ad3_022 | 2 | Saliva | 5 |  |
| 141128-ad3_023 | 2 | Saliva | 6 |  |
| 141128-ad3_025 | 2 | Saliva | 7 |  |
| 141128-ad3_027 | 1 | Saliva | 6 |  |
| 141128-ad3_028 | 1 | Saliva | 5 |  |
| 141128-ad3_029 | 2 | Saliva | 8 |  |
| 141128-ad3_030 | 1 | Saliva | 7 |  |
| 141128-ad3_031 | 1 | Saliva | 8 |  |
| 141128-ad3_032 | 2 | Saliva | 9 |  |
| 141128-ad3_033 | 1 | Saliva | 9 |  |
| 141128-ad3_034 | 1 | Saliva | 10 |  |
| 150402-ad044_S42 | Kontrolle-Schmelz | Kontrolle-Schmelz |  |  |
| 150402-ad045_S43 | Kontrolle-Schmelz | Kontrolle-Schmelz |  |  |
| 150402-ad046_S44 | Kontrolle-Schmelz | Kontrolle-Schmelz |  |  |
| 150402-ad047_S45 | Kontrolle-PCR | Kontrolle-PCR |  |  |
| 150402-ad048_S46 | Kontrolle-NTC | Kontrolle-NTC |  |  |

**Supplementary Table 2 (please open supplementary dataset)**: Table with all OTUs present in samples and controls. The table contains the following information: OTU ID (column “OTU”); taxonomic lineage assigned by LotuS (columns “Kingdom” to “Species”); top hit of the BLAST search in eHOMD with the corresponding taxon (column “eHOMD”), match identity (column “eHOMDindent”) and mismatches (column “eHOMDmismatch”); a column containing a flag showing whether the OTU is contain in any control sample (column “inControls”, the value is “Yes” if present in any control); OTU counts per sample (all remaining columns).

**Supplementary Tables 3**: Overview of the OTUs with significant differences in their abundance in CC and NC sample groups considering 2 h (a), 4 h (b), 8 h (c), and saliva (d) group separately. The table contains the OTU ID (column “otu”), WMW test statistic (column “wmw_statistic”), log fold-change (bases 2, column “log2_fc”), the raw and adjusted p-values (columns “p” and “adj_p”), and the AUC (column “auc”). A positive log fold-change indicates higher presence in CC samples.

| log2_fc: (-) signifikant for no cavitated caries |
| --- |
| log2_fc: ( ) signifikant for cavitated caries |

(a)

| **otu** | **wmw_statistic** | **log2_fc** | **P** | **auc** | **adj_p** |  |
| --- | --- | --- | --- | --- | --- | --- |
| OTU_624 | 65 | Inf | 0.045323 | 0.642857 | 0.495585 | |
| OTU_494 | 52 | 3.47231341753159 | 0.020683 | 0.785714 | 0.495585 | |
| OTU_243 | 126 | -Inf | 0.013510 | 0.307692 | 0.495585 | |
| OTU_77 | 52 | Inf | 0.010274 | 0.714285 | 0.495585 | |
| OTU_363 | 127.5 | -3.45589516760706 | 0.023413 | 0.299450 | 0.495585 | |
| OTU_97 | 59 | 3.63331303333882 | 0.047334 | 0.675824 | 0.495585 | |

(b)

| **otu** | **wmw_statistic** | **log2_fc** | **P** | **Auc** | **adj_p** |
| --- | --- | --- | --- | --- | --- |
| OTU_71 | 143 | -1.04931058037706 | 0.003927 | 0.214285 | 0.484845 |
| OTU_134 | 65 | Inf | 0.045323 | 0.642857 | 0.484845 |
| OTU_290 | 65 | Inf | 0.045323 | 0.642857 | 0.484845 |
| OTU_253 | 119 | -Inf | 0.030878 | 0.346153 | 0.484845 |
| OTU_1022 | 119 | -Inf | 0.030878 | 0.346153 | 0.484845 |
| OTU_633 | 65 | Inf | 0.045323 | 0.642857 | 0.484845 |
| OTU_39 | 36 | 6.28660436202286 | 0.002273 | 0.802197 | 0.484845 |
| OTU_1496 | 119 | -Inf | 0.030878 | 0.346153 | 0.484845 |
| OTU_826 | 65 | Inf | 0.045323 | 0.642857 | 0.484845 |
| OTU_144 | 53 | 2.4333376249777 | 0.045657 | 0.708791 | 0.484845 |
| OTU_198 | 130 | -3.8856263837612 | 0.040185 | 0.285714 | 0.484845 |
| OTU_174 | 128 | -3.35466253040414 | 0.034802 | 0.296703 | 0.484845 |

(c)

| **otu** | **wmw_statistic** | **log2_fc** | **P** | **Auc** | **adj_p** |
| --- | --- | --- | --- | --- | --- |
| OTU_201 | 53.5 | 4.8592818349925 | 0.0323898 | 0.706043 | 0.531993 |
| OTU_447 | 58.5 | Inf | 0.0219620 | 0.678571 | 0.531993 |
| OTU_94 | 51 | 4.36403671725705 | 0.0312997 | 0.719780 | 0.531993 |
| OTU_103 | 134 | -4.2277062879714 | 0.0235114 | 0.263736 | 0.531993 |
| OTU_295 | 58.5 | Inf | 0.0219620 | 0.678571 | 0.531993 |
| OTU_640 | 58.5 | Inf | 0.0219620 | 0.678571 | 0.531993 |
| OTU_137 | 53 | 3.44238199512696 | 0.0409322 | 0.708791 | 0.531993 |
| OTU_314 | 45.5 | Inf | 0.0046073 | 0.75 | 0.531993 |
| OTU_472 | 45.5 | Inf | 0.0046073 | 0.75 | 0.531993 |
| OTU_837 | 65 | Inf | 0.0453235 | 0.642857 | 0.531993 |
| OTU_832 | 65 | Inf | 0.0453235 | 0.642857 | 0.531993 |
| OTU_1007 | 58 | 4.19137584672285 | 0.0407312 | 0.681318 | 0.531993 |
| OTU_474 | 65 | Inf | 0.0453235 | 0.642857 | 0.531993 |
| OTU_83 | 42 | 5.74410153008783 | 0.0097439 | 0.769230 | 0.531993 |
| OTU_166 | 52 | 3.65084509352905 | 0.0358408 | 0.714285 | 0.531993 |
| OTU_915 | 65 | Inf | 0.0453235 | 0.642857 | 0.531993 |
| OTU_77 | 43.5 | 4.94997716657573 | 0.0139796 | 0.760989 | 0.531993 |
| OTU_932 | 58.5 | Inf | 0.0219620 | 0.678571 | 0.531993 |
| OTU_112 | 46 | 2.63824104797893 | 0.0286956 | 0.747252 | 0.531993 |
| OTU_39 | 48 | 1.97071223369252 | 0.0385755 | 0.736263 | 0.531993 |
| OTU_585 | 59 | 5.36992760154414 | 0.0473346 | 0.675824 | 0.531993 |
| OTU_308 | 50 | 5.26396795095666 | 0.0149372 | 0.725274 | 0.531993 |
| OTU_42 | 40 | 3.71397469521787 | 0.0093435 | 0.780219 | 0.531993 |
| OTU_145 | 133 | -5.37083918938089 | 0.0164047 | 0.269230 | 0.531993 |
| OTU_68 | 34 | 2.33346410709322 | 0.0056841 | 0.813186 | 0.531993 |
| OTU_436 | 58.5 | Inf | 0.0219620 | 0.678571 | 0.531993 |
| OTU_755 | 58.5 | Inf | 0.0219620 | 0.678571 | 0.531993 |
| OTU_246 | 58.5 | Inf | 0.0219620 | 0.678571 | 0.531993 |
| OTU_58 | 42.5 | 1.40723320874904 | 0.0182894 | 0.766483 | 0.531993 |
| OTU_157 | 149 | -3.64109041226492 | 0.0030812 | 0.181318 | 0.531993 |
| OTU_254 | 57 | 5.32989233100857 | 0.0349258 | 0.686813 | 0.531993 |

(d)

| **otu** | **wmw_statistic** | **log2_fc** | **P** | **Auc** | **adj_p** |
| --- | --- | --- | --- | --- | --- |
| OTU_134 | 142 | -1.54237038573542 | 0.012467 | 0.219780 | 0.678748 |
| OTU_290 | 26 | Inf | 0.000303 | 0.857142 | 0.151655 |
| OTU_250 | 48 | 1.56746775024577 | 0.036678 | 0.736263 | 0.678748 |
| OTU_935 | 125.5 | -2.48961140167095 | 0.032298 | 0.310439 | 0.678748 |
| OTU_1092 | 122 | -4.68787457550199 | 0.042035 | 0.329670 | 0.678748 |
| OTU_1039 | 119 | -Inf | 0.030878 | 0.346153 | 0.678748 |
| OTU_1648 | 122 | -2.74273050270994 | 0.042035 | 0.329670 | 0.678748 |
| OTU_239 | 126 | -7.29461672628794 | 0.038142 | 0.307692 | 0.678748 |
| OTU_380 | 143 | -1.31411621143186 | 0.011986 | 0.214285 | 0.678748 |
| OTU_1776 | 58.5 | Inf | 0.021962 | 0.678571 | 0.678748 |
| OTU_278 | 44.5 | 0.905332582560492 | 0.023735 | 0.755494 | 0.678748 |
| OTU_625 | 128 | -2.97695007662644 | 0.040957 | 0.296703 | 0.678748 |
| OTU_291 | 135 | -1.38985478365878 | 0.034229 | 0.258241 | 0.678748 |
| OTU_1575 | 126 | -2.29877201861128 | 0.038142 | 0.307692 | 0.678748 |
| OTU_270 | 138.5 | -1.55683092646009 | 0.022358 | 0.239010 | 0.678748 |
| OTU_256 | 142 | -0.584552078464772 | 0.014248 | 0.219780 | 0.678748 |
| OTU_1276 | 146 | -1.51735178459151 | 0.005635 | 0.197802 | 0.678748 |
| OTU_77 | 49 | 3.55770499158559 | 0.043704 | 0.730769 | 0.678748 |
| OTU_1062 | 133.5 | -2.24288482516384 | 0.022049 | 0.266483 | 0.678748 |
| OTU_1118 | 39 | Inf | 0.001968 | 0.785714 | 0.491121 |
| OTU_632 | 50.5 | 2.94373088620411 | 0.036462 | 0.722527 | 0.678748 |
| OTU_1569 | 127.5 | -2.83670987726995 | 0.023413 | 0.299450 | 0.678748 |
| OTU_1531 | 124.5 | -2.1633890267602 | 0.037733 | 0.315934 | 0.678748 |
| OTU_216 | 133 | -2.82787248405425 | 0.012631 | 0.269230 | 0.678748 |
| OTU_719 | 128.5 | -2.91807319219105 | 0.019828 | 0.293956 | 0.678748 |
| OTU_755 | 49 | 1.36720991931822 | 0.037063 | 0.730769 | 0.678748 |
| OTU_244 | 46 | 2.20717326135212 | 0.029397 | 0.747252 | 0.678748 |
| OTU_568 | 58.5 | Inf | 0.021962 | 0.678571 | 0.678748 |
| OTU_157 | 132 | -0.685348825674922 | 0.034185 | 0.274725 | 0.678748 |
| OTU_254 | 43 | 1.18531219647737 | 0.020074 | 0.763736 | 0.678748 |

**Supplementary Table 4 (please open supplementary dataset)**: Absolute differences in correlation values of mean OTU abundances against time points between individuals with and without cavitated caries. The absolute difference in the correlation values for the groups (CC vs. NC) was computed, possibly reaching a maximal value of 2.
